# Functional Analysis of TAAR1 Expression in the Intestine Wall and the Effect of Its Gene Knockout on the Gut Microbiota in Mice

**DOI:** 10.3390/ijms252313216

**Published:** 2024-12-09

**Authors:** Anastasia N. Vaganova, Ilya S. Zhukov, Taisiia S. Shemiakova, Konstantin A. Rozhkov, Lyubov S. Alferova, Alena B. Karaseva, Elena I. Ermolenko, Raul R. Gainetdinov

**Affiliations:** 1 Institute of Translational Biomedicine, St. Petersburg State University, Universitetskaya nab. 7/9, St. Petersburg 199034, Russia; 2 St. Petersburg State University Hospital, St. Petersburg State University, Universitetskaya nab. 7/9, St. Petersburg 199034, Russia; 3 Federal State Budgetary Scientific Institution «Institute of Experimental Medicine» (FSBSI «IEM»), Str. Academica Pavlova 12, St. Petersburg 197022, Russia

**Keywords:** trace amine-associated receptor, TAAR, TAAR1, gut microbiota, transcriptomic data, colon, intestine, enteroendocrine cells, myenteric neurons

## Abstract

Currently, the TAAR1 receptor has been identified in various cell groups in the intestinal wall. It recognizes biogenic amine compounds like phenylethylamine or tyramine, which are products of decarboxylation of phenylalanine and tyrosine by endogenous or bacterial decarboxylases. Since several gut bacteria produce these amines, TAAR1 is suggested to be involved in the interaction between the host and gut microbiota. The purpose of this present study was to clarify the TAAR1 function in the intestinal wall and estimate the TAAR1 gene knockout effect on gut microbiota composition. By analyzing public transcriptomic data of the GEO repository, we identified TAAR1 expression in enterocytes, enteroendocrine cells, tuft cells, and myenteric neurons in mice. The analysis of genes co-expressed with TAAR1 in enteroendocrine cells allows us to suggest the TAAR1 involvement in enteroendocrine cell maturation. Also, in myenteric neurons, we identified the co-expression of TAAR1 with calbindin, which is specific for sensory neurons. The 16S rRNA gene-based analysis of fecal microbiota revealed a slight but significant impact of TAAR1 gene knockout in mice on the gut microbial community, which manifests in the higher diversity, accompanied by low between-sample variability and reorganization of the microbial co-occurrence network.

## 1. Introduction

G protein-coupled trace amine-associated receptors (TAARs) recognize both biogenic monoamine and polyamine ligands. Six functional TAARs, including TAAR1, TAAR2, TAAR5, TAAR6, TAAR8, and TAAR9, were recognized in humans [1,2], and TAAR1 is the most studied receptor in this family to date. TAAR1 was identified in the colonic epithelium, both in the cytoplasmic membrane and in the intracellular compartment [1,3,4] of different cell types. In particular, this receptor is expressed in enterocytes [5,6,7], immune cells [8,9], myenteric neurons [3], enteric glia [4], and intestinal neuroendocrine cells [4,8].

Today, the development of TAAR-based therapies is one of the breakthrough neuropharmacological fields of study. Significant effects of TAAR1 agonists were demonstrated in the central nervous system. [2,10]. Ulotaront (SEP-363856) and Ralmitaront (RO6889450) have already been investigated in phase II clinical trials to treat schizophrenia, and Ulotaront is currently being tested in phase III clinical trials to treat schizophrenia, major depression, and generalized anxiety disorder [11,12]. However, TAAR1 was found outside the central nervous system, and its impact on glycemic control, biochemical composition of the blood, and chemosensory regulation was demonstrated [13,14,15,16,17].

TAARs’ ligands, trace amines (TA), are a group of biogenic amines that are contained in the mammalian central nervous system in trace amounts, i.e., in nanomolar concentrations. However, these compounds are synthesized not only by endogenous mammalian decarboxylases but also by the gut microbiota through the decarboxylation of amino acids and their concentrations in feces, reaching 5–10 μg/g [2,18]. They represent a group of ligands for the TAAR receptor family that potentially may be involved in the brain–gut-microbiome axis interaction.

Numerous natural biogenic amines activate TAAR1, but beta-phenylethylamine (PEA) and tyramine (TYR) are currently considered the most prominent of them [2]. In the gastrointestinal tract, TAAR1 agonists stimulate motility [3,4,19,20,21], release neuropeptide Y and monoamine neurotransmitters [4], modulate immune landscape and inflammatory signaling [22,23], increase the synthesis of serotonin by enteroendocrine cells [4,6,24,25,26], decrease the permeability of the gut wall, and increase the water content in feces and colonic secretion [7]. Also, TYR can promote the adherence of microbes to the intestinal epithelial cells [4].

**Figure 1:**
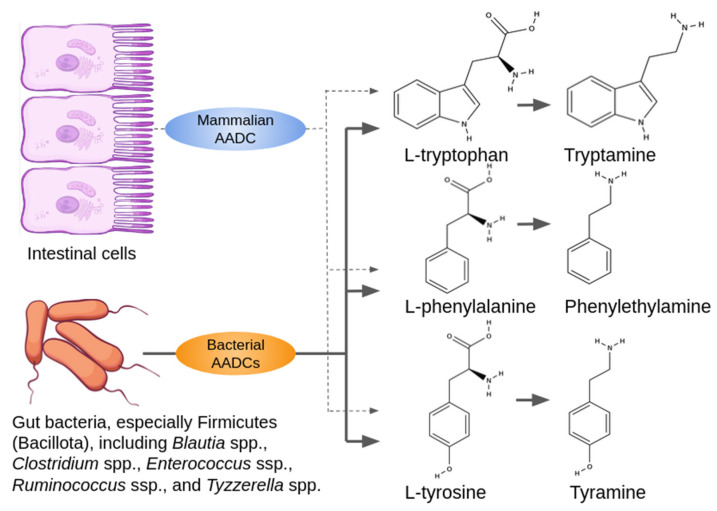
Sources of trace amines (TA) in the gut. The major process of TA biogenesis marked by the bold arrows is the decarboxylation of amino acids by bacterial aromatic amino acid decarboxylases (AADCs). Additionally, mammalian AADC (encoded by gene *DOPA decarboxylase*, *DDC*) is produced by intestine epithelial cells involved in the TA biogenesis in the gastrointestinal tract as marked by dotted arrows.

The human small intestine cells express genes involved in the metabolism of trace amines, including *ACE2* and *SCL6A19*, which allow for the influx of L-tryptophan and phenylalanine from the intestinal lumen; AADC (encoded by *DDC* gene) converting L-tryptophan, tyrosine, and phenylalanine into tryptamine (TRY), TYR, and PEA (Figure 1), respectively; and MAO-B, which degrades trace amines [27]. However, microbiota are suggested to be the primary source of trace amines in the gut. For example, the genetic metabolic disease characterized by the absence of trace amine trimethylamine (TMA) degradation, with an associated foul-smelling body odor, is treatable by broad-spectrum non-absorbed antibiotics such as neomycin, so the origin of TMA is presumed to be in the microbiome [28].

Decarboxylation of L-amino acids, accompanied by trace amine production, has been shown to occur during putrefaction of tissue, be involved in bacterial fermentation of certain foodstuffs, and is generally considered a strategy for certain bacteria to adapt to acidic environmental conditions [2,4,29]. Several representatives of the phylum Firmicutes (Bacillota), including genera *Blautia*, *Clostridium*, *Enterococcus*, *Ruminococcus*, and *Tyzzerella*, which are dominant gut bacteria, produce aromatic amines like PEA, TYR, and TRY by acting aromatic amino acid decarboxylase (AADC) (Figure 1). Bacterial AADCs decarboxylate different amino acids, whose spectrum varies between species [6,29]. Some AADCs also could decarboxylate L-DOPA [6]. Staphylococcal aromatic amino acid decarboxylase (SadA) decarboxylates 5-hydroxytryptophan (5-HTP) into serotonin; L-DOPA to dopamine; and produces TRY, TYR, and PEA by decarboxylation of tryptophan, tyrosine, and phenylalanine, respectively [8,24,30]. At the same time, tryptophan can be converted into TRY by specific decarboxylases in *Clostridium sporogenes* [8] or phylogenetically distinct enzymes of *Ruminococcus gnavus* [24,31], and tyrosine is decarboxylated to TYR by the bacterial tyrosine decarboxylase (TyrDC) and exported from the microorganism by the tyrosine transporter TyrP [4]. So, gut microbiota produces neuroactive ligands such as trace amines, serotonin, or dopamine in the same pathway [32,33].

Changes in gut microbiota composition accompany gastrointestinal diseases, such as inflammatory bowel syndrome, which is associated with increased amino acid catabolism and higher levels of polyamines [25]. Elevated concentrations of the major TAAR1 ligands, TYR and PEA, were identified in celiac disease, colorectal cancer, and inflammatory bowel disease [4,24,25,34,35,36]. TYR and PEA induce gastrointestinal transit acceleration and colonic secretion mediated by 5-HT production [7,8,18]. The effect of these amines’ overproduction also may be systemic; for example, TYR or PEA significantly suppresses the insulin-induced Akt phosphorylation in major metabolic tissues, including white adipose tissue, liver, and skeletal muscle, which leads to the development of insulin resistance and type 2 diabetes [18]. TAAR1 mediates both the gastrointestinal and systemic effects of trace amine excess in inflammatory bowel disease [7].

Taking into consideration the described involvement of TAAR1 in the interaction between the gut microbial community and host, we studied the impact of TAAR1 knockout on the mouse gut microbiota composition. In addition, in view of the previously published data for TAAR1 expression in the gut wall, we also analyzed TAAR1 co-expressed gene profiles in public transcriptomic data to identify functional relationships of TAAR1 in cells isolated from intestine samples.

## 2. Results

### 2.1. TAAR1 Expression Is Heterogeneously Distributed in Colon Epithelium Cell Population

To summarize and quantify the current knowledge of TAAR1 gene expression in different cell populations in the mouse intestinal wall, we searched the Gene Expression Omnibus (GEO) database for the relevant mouse datasets as described in the Materials and Methods Section 4.1. The datasets included in the analysis are detailed in Table 1.

**Figure 2:**
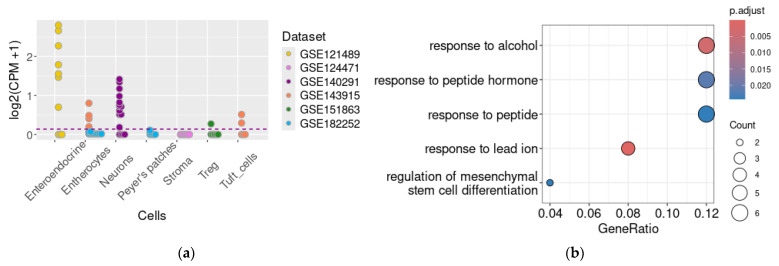

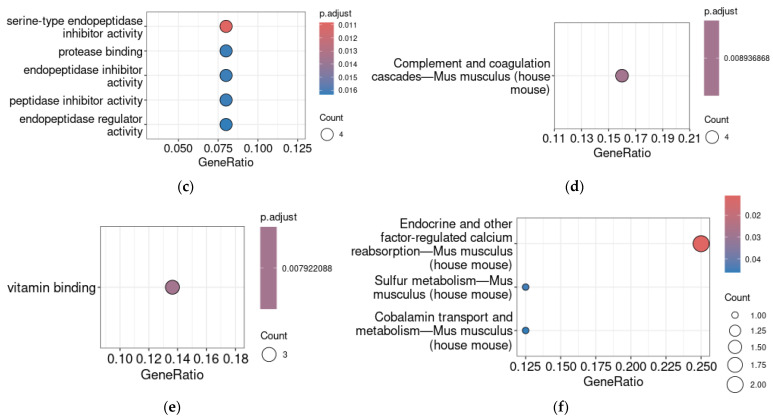
TAAR1 mRNA expression in the fractionated murine intestine wall cell samples. The expression of TAAR1 mRNA in various cell fractions described in different datasets is represented. Threshold level (0.1 CPM) is marked with a dotted line (**a**). Functional analysis of top 50 genes co-expressed (cut-off values were ρ > 0.7, *p* < 0.05) with TAAR1 in normal adult wild-type mice enteroendocrine cells (GSE121489) with Gene Ontology (GO) Biologic process (BP) term enrichment analysis, (**b**) GO molecular function (MF) term enrichment analysis (**c**), or Kyoto Encyclopedia of Genes and Genomes (KEGG)-pathway enrichment analysis (**d**). Functional analysis of genes (*n* = 26) co-expressed with TAAR1 in enteric neurons (cut-off values were ρ > 0.7, *p* < 0.05, GSE140291) by (**e**) GO MF term enrichment analysis or (**f**) KEGG pathway enrichment analysis.

The study of transcriptomic RNA-generated datasets showed that *TAAR1* mRNA is expressed in the enteroendocrine cells (GSE121489, 7/10 samples, 70%) in both the ileum and colon samples (Figure 2a). The differential expression analysis does not reveal significant differences in *TAAR1* mRNA level between the samples originating from the ileal or colon part. *TAAR1* mRNA was also identified in GSE143915 in RNA samples isolated from the tuft cells, which is a rare and understudied group of gut epithelial cells with specific chemosensory and secretory capacities [37].

The nuclear fraction of neuronal cells extracted from the intestinal wall also contains *TAAR1* mRNA (GSE140291, 12/16 samples, 75%). Additionally, the analysis of the GSE143915 dataset identified TAAR1 mRNA in epithelial cell transcriptomes, with representation in four out of five samples (80%). However, these results were not replicated in the analysis of another dataset, GSE182252, where all enterocyte samples either tested negative for TAAR1 mRNA or expressed it below the cut-off level (Figure 2a), i.e., represented *TAAR1* normalized expression levels below 0.1 CPM (counts per million).

No *TAAR1* transcripts were identified in the regulatory T cell data represented in the GSE15186 dataset, except in a single sample (Figure 2a).

### 2.2. Genes Co-Expressed with TAAR1 in the Enteric Secretory Cells

For further analysis of TAAR1 function in the enteric secretory cells, we analyzed genes whose expression levels correlated with *TAAR1* mRNA expression in the GSE121489 dataset, representing *TAAR1* expression in the enteroendocrine cells. Genes were selected using Spearman’s correlation coefficient (cut-off values were ρ > 0.7, *p* < 0.05).

*TAAR1* mRNA co-expressed gene clusters identified as described in the Materials and Methods included 1383 genes; thus, only the top 50 co-expressed genes (Supplementary Table S1) of this cluster were selected for further analysis. The Gene Ontology Biologic process (GO BP) enrichment analysis identified that six of fifty of the most significantly *TAAR1*-co-expressed genes (i.e., 12% of the analyzed gene cluster) are involved in response to alcohol, peptides, or peptide hormones (Figure 2b). The statistically strongest association, identified by GO BP enrichment analysis, is the co-expression of *TAAR1* with genes involved in the response to lead ions. Such enrichment results are associated with high *TAAR1* co-expression with *Serpina1a–c* and *Serpina1e*, which are related to all four GO terms. The association of *TAAR1*-co-expressed genes with the GO molecular function (MF) terms “serine-type endopeptidase inhibitor activity”, “protease binding”, “endopeptidase inhibitor activity”, and “peptidase inhibitor activity” is determined by the co-expression of TAAR1 with the same serpin genes, as revealed by the GO MF enrichment test (Figure 2c). The Kyoto Encyclopedia of Genes and Genomes (KEGG)-pathway enrichment analysis test identified the co-expression of *TAAR1* with genes involved in complement and coagulation cascades. Here, the enrichment core also comprises four genes, i.e., *Serpina1a–c* and *Serpina1e* (Figure 2d). So, these four genes seem to be the most functionally interconnected in the top 50 of *TAAR1* co-expressed genes in enteroendocrine cells.

### 2.3. Genes Co-Expressed with TAAR1 in the Myenteric Neural Cells

By the same approach as described above, we selected genes whose expression levels correlated with *TAAR1* mRNA expression in the GSE140291 dataset, which consists of transcriptomic data for the enteric neurons. Genes were selected by Spearman’s correlation coefficient (cut-off values were ρ > 0.7, *p* < 0.05).

The identified *TAAR1* co-expressed gene cluster is significantly narrower in neural cells compared to enteroendocrine cells. It includes only 26 genes annotated in the Gene NCBI database.

No significant results were acquired by the GO BP enrichment test in the represented gene cluster. The GO MF and KEGG pathway enrichment analysis revealed similar patterns. In both tests, the positive results, i.e., enrichment with genes characterized by the term “vitamin binding” (Figure 2e) or involved in the “Endocrine and other factor-regulated calcium reabsorption—Mus musculus (house mouse)” pathway (Figure 2f), respectively, mirror the co-expression of *TAAR1* with calbindin (*Calb1*) and vitamin D (1,25-dihydroxy vitamin D3) receptor (*VDR*). Additionally. Transcobalamin II (*Tcn2*), characterized by the GO term “vitamin binding”, is also co-expressed with TAAR1 in enteric neurons.

In the neural cells, *TAAR1* likewise is co-expressed with several genes of DNA-binding proteins and receptors, but the applied enrichment test did not identify any common functions for these genes (Supplementary Table S2).

### 2.4. Gut Microbiota Composition in TAAR1 Knockout Mice

Since *TAAR1* expression was previously demonstrated in the different cell groups in the gut wall, we attempted to estimate its knockout effect on the gut microbial community. For this purpose, we collected feces samples from mice with knock-outed *Taar1* gene (TAAR1-KO mice, *n* = 10) and their WT littermates (*n* = 10). The fecal microbiota diversity was estimated using 16 S rRNA high-throughput sequencing.

**Figure 3:**
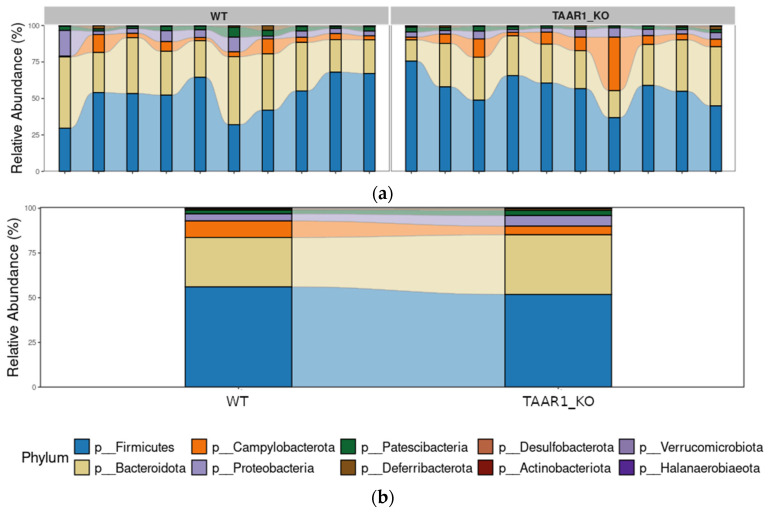
The basic structure of the bacterial community composition. Relative abundance of fecal microbiota composition at the phylum level in each sample (**a**) and study group (**b**) for TAAR1-KO mice and wild-type littermates. The top ten phyla are presented. Each bar in (**a**) represents a single fecal sample, and each bar in (**b**) represents a group.

As a result of the analysis of mouse fecal samples, including 10 samples from TAAR1-KO mice and 10 samples from their WT littermates, a total of 108,808 unique reads were obtained, which corresponded to 107 genera of bacteria. Altogether, thirteen bacterial phyla were detected, of which four phyla were the most abundant, including Firmicutes (53.9%, SD = 12.15%), Actinobacteria (Actinomycetota) (30,5%, SD = 8.80%), Proteobacteria (Pseudomonadota) (4.9%, SD = 3.6%), and Campylobacterota (2.1%, SD = 3.5%) (Figure 3a,b).

When comparing WT samples with the samples received from their TAAR1-KO littermates, no significant differences in the abundance of any phyla were identified. Additionally, we found no significant differences for any identified class, order, family, or genus.

### 2.5. Alterations in Fecal Microbiota α-Diversity in TAAR1-KO Mice

**Figure 4:**
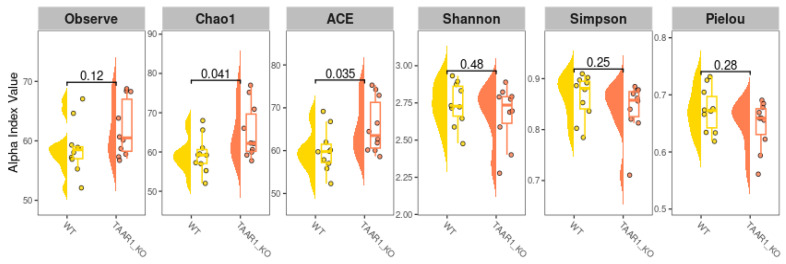
The comparison of gut microbiota α-diversity among TAAR1-knockout (TAAR1-KO) and wild-type (WT) mice. α-diversity was measured by observed OTUs, Chao1, abundance-based coverage estimator (ACE), Shannon, Simpson, and Pielou. Box plots and violin plots depict microbiome diversity and abundance differences according to each test. The horizontal line inside the box represents the median. Individual sample values are represented by dots. Wilcoxon’s test was applied to compare groups.

An α-diversity measures the variation in a single sample, so we compared different α-diversity metrics between TAAR1-KO and WT fecal samples (Figure 4). The mean observed OTU numbers were not statistically different between the two groups. The Chao1 and abundance-based coverage estimators (ACE) estimate the community richness by adding a correction factor to the observed number of species. For both of these parameters, the mean value of α-diversity is higher in the TAAR1-KO group (*p* < 0.05) Shannon index and Simpson index, which were calculated considering both richness (the number of species) and evenness (how evenly species are represented), and did not demonstrate significant differences between the two genotypes. Pielou’s evenness also does not differ between groups.

### 2.6. The TAAR1-KO Genotype Is Associated with More Stable Fecal Microbiome Composition and Slight Reorganization of Bacterial Interactions in the Gut Microbial Community

**Figure 5:**
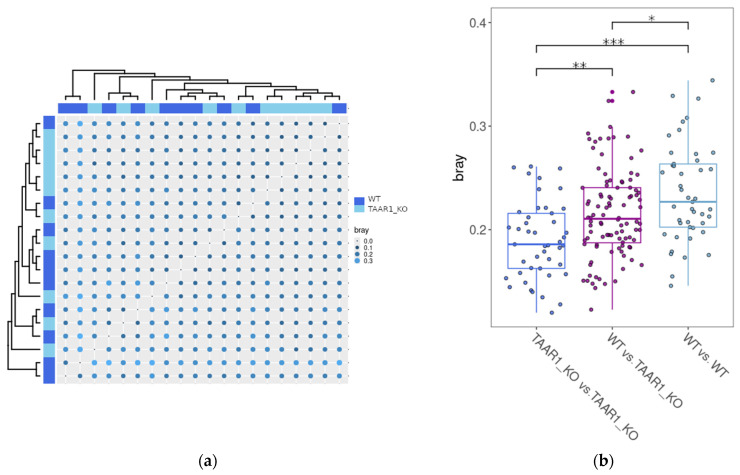
Heatmap (**a**) representing the Bray–Curtis dissimilarity (bray) in the bacterial community structure between samples. The genotype is marked by color. No clustering of samples based on the genotype (i.e., WT or TAAR1-KO) was identified. Box plots (**b**) of the Bray–Curtis dissimilarity index within groups and between groups represent significant differences between phenotypes. Wilcoxon’s test was applied to compare Bray–Curtis dissimilarity values in TAAR1-KO and WT groups. These results confirm the differences in β-diversity among TAAR1-KO and wild-type (WT) mice. *—*p* value < 0.05, **—*p* value < 0.01, ***—*p* value < 0.001.

To evaluate the β-diversity of gut microbiota in TAAR1-KO mice and their WT littermates, we estimated the between-sample differences using the Bray–Curtis dissimilarity test. As represented in the heat map (Figure 5a), beta diversity values did not rearrange samples into different clusters that mirror the host genotype. The heat map (Figure 5a) shows that TAAR1-KO and WT samples cluster together, and samples from mice with the same genotype may be positioned on distant clades.

However, when we compared the Bray–Curtis distance distribution among the groups, we identified that the WT mice-derived samples exhibited more heterogeneity and dissimilarity from one another compared to the TAAR1-KO group (Figure 5b).

Co-occurrence analysis for bacterial families identified 630 possible pairs of families in WT samples and 666 pairs of families in TAAR1-KO samples (mitochondrial 16 S RNA was included in the analysis). Among the pairs identified in WT mice, no pairs occurred in the same host more or less frequently than expected. In contrast, in TAAR1-KO mice, *Eggerthellaceae* family representatives more frequently co-occurred with *Ethanoligenenaceae* than expected (*p* = 0.024).

The visualization of the identified bacterial family co-occurrence network topology in WT (Supplementary Figure S1) and TAAR1-KO (Supplementary Figure S2) identified that the network topology depends on the host genotype (the pairs with expected co-occurrence < 1 and families represented by <5 counts in OTU table were removed from the analysis).

## 3. Discussion

Previously, TAAR1 expression was identified in different groups of intestinal wall cells [1], including the enterocytes [5,6,7], intestinal neuroendocrine cells [4,8], myenteric neurons [3], and glia [4], as well as immune cells [1,8,9]. Taking into account the different origins of these cell groups and the biological distinctions between them, we attempted to estimate *TAAR1* expression and functional associations in different cell fractions by an analysis of public transcriptomic data. Additionally, we evaluated if *TAAR1* expression in the intestinal epithelium cells may be significant for the interaction between the gut microbial community, which commonly includes several trace amine producers [38,39], and the host macroorganism. To confirm this suggestion, we studied microbiota composition in the fecal samples of TAAR1-KO mice and their WT littermates.

According to the previously published data [3,4,5,6,7], the analysis of public transcriptomic data allows us to demonstrate *TAAR1* expression in the enterocytes, enteroendocrine, and neural cells. Additionally, we show that *TAAR1* mRNA is expressed in the tuft cells by the same approach. Tuft cells are a specific rare cell population that express taste and succinate receptors [37]. It seems expected that TAAR1 is expressed in these cells, as previous studies have identified *TAAR1* co-expression with chemosensory receptors in other tissues such as pancreatic islets [40], which are known for their complex repertoire of chemosensory receptors, or in benign melanocytes in nevi [16]. In this study, we could not provide a more detailed analysis of TAAR1’s functional role in enterocytes or tuft cells because of the insufficient number of available samples for the co-expressed gene identification.

We identified TAAR1 expression in enteroendocrine cells isolated from mouse ileal and colonic samples and found a significant association between Serpina1a–c and e and TAAR1 expression in enteroendocrine cell samples that were included in the analysis. Serine protease inhibitors, also known as serpins, demonstrate anti-inflammatory properties; serve as hormone transporters, chaperones, and antiangiogenic factors; and participate in blood coagulation, insulin sensitivity, and tissue remodeling. In the gastrointestinal tract, serpins, including Serpina1, support the proteolytic balance [41,42]. Previous studies have described Serpina1 expression in gut enteroendocrine cells, starting during enteroendocrine cell maturation and remaining at lower levels in mature cells [43,44]. Thus, the identified co-expression of *TAAR1* with *Serpina1* genes may mirror TAAR1 expression upregulation in premature enteroendocrine cells.

The associations between TAAR1 expression and other genes in neural cells are less pronounced and represent only weak functional relations because of the high heterogeneity of this cell population [45]. Calbindin, which is co-expressed with TAAR1 in this cell group, is expressed among the distinct types of mesenteric neurons [46] and is especially associated with intrinsic sensory neurons and some interneurons [47]. It was demonstrated that the number of calbindin-positive neurons decreases after the vancomycin-induced deviations in microbiota abundance in adolescence, accompanied by slower colonic migrating motor complexes [48]. In contrast, similar treatment in neonatal mice induces colon motility and increases the number of calbindin-positive neurons [47]; however, no differences in calbindin expression were identified in mesenteric neurons of specific pathogen-free and germ-free mice [48].

Insufficient information is available on the function of the other two genes involved in vitamin binding and co-expressed with TAAR1 in mesenteric neurons. VDR is expressed in different classes of neural cells [49] but was not revealed in the mesenteric neurons previously [50]. Transcobalamin 2 expression was demonstrated in colon tissue [51] but no data for its expression in different cell types are currently available.

Intestinal stromal cells are mesenchymal cells that support the epithelium and participate in epithelial cell differentiation and immune response regulation [52]; no TAAR1 expression was identified in this population in this present study. Additionally, this study did not identify TAAR1 expression in regulatory T cells. Previously, TAAR1 was identified in T cells [9], so the negative result in our study may be related to the insufficient sequencing depth of RNAseq data included in the analysis. Similarly, this source of false-negative results is not excluded in all other cases, in which we did not reveal any TAAR1 expression.

By the study of fecal microbiome composition in TAAR1-KO mice and their WT littermates, we found that the representative abundance of Firmicutes and Bacteroidota is predominant in both groups, as has been previously identified in other studies of WT mice [53,54]. Phylum Proteobacteria and Campylobacterota, previously known as Epsilonproteobacteria [55], which are also well-known components of the gut microbial community, were the third and fourth most abundant phyla identified in studied fecal samples.

The differential abundance analysis did not reveal any taxa that could be considered more specific to the TAAR1-KO mouse fecal microbiota or WT littermates. Meanwhile, we identified differences in the gut microbial community between the two groups when comparing α- and β-diversity characteristics. The fecal microbiota of TAAR1-KO mice demonstrates slightly higher α-diversity than in WT mice. Such a difference is difficult to interpret as some benefit or disadvantage in the interaction between host and gut microbial community. Commonly, higher gut microbiota diversity is associated with better health and well-being; for example, higher α-diversity was identified in samples from individuals with a smaller body mass index compared to subjects with obesity [56], or in persons with better emotional background [57]. However, high gut microbiota α-diversity in infants may be associated with a higher risk of respiratory infections or diarrhea [58]. Also, the examination of gut microbiota in adults with type 1 diabetes showed higher α-diversity than in the healthy control group [59], and the same result was demonstrated when patients with IBS were compared with healthy controls [60].

Heat map analysis examining β-diversity (Figure 5a) did not reveal clustering that mirrors the mice genotype, i.e., that clearly separates WT samples from the TAAR1-KO ones. However, the knockout of TAAR1 leads to the development of a more stable microbiota profile, as evidenced by lower values of Bray–Curtis dissimilarity. Interestingly, the opposite destabilizing effect of TAAR9 knockout on the gut microbiota composition was previously revealed in rats [61]. These findings suggest that TAAR1 knockout had an apparent effect on β-diversity.

Additionally, we identified a slight but significant impact of TAAR1 knockout on the interaction of gut microbial community members. First, we observed the onset of co-occurrence between *Eggerthellaceae* family representatives and *Ethanoligenenaceae* in TAAR1-KO fecal samples. *Ethanoligenenaceae* family members are fermenters that produce ethanol and hydrogen [62,63]. Family *Eggerthellaceae* consists of bacteria, which hydrolyze amino acids such as arginine and leucine [64], whose activity grows in the presence of hydrogen-producing helper bacteria [65]. The enhancement of the association between *Ethanoligenenaceae* and *Eggerthellaceae* in TAAR1-KO together with the reorganization of bacterial family co-occurrence network topology may reflect some features of the intestine’s internal environment that are specific to TAAR1-KO and impact microbiota well-being.

The findings of this study must be seen while considering some limitations. The recently published data confirm that fecal microbiome composition is insufficient in representing the whole gut microbiome [66,67]. Also, it was revealed that the abundance of Firmicutes gradually decreased from the stomach to feces while the abundance of Bacteroidetes gradually increased [66]. On the other hand, the major producers of TAAR1 ligand TYR, like *Blautia hansenii*, *Clostridium* spp., or *Ruminococcus gnavus* [68,69], as well as PEA-producing strains of *Enterococcus* spp. [70], belong to the phyla Firmicutes. Also, the studied mice received a standard diet; thus, the estimation of the TAAR1 gene knockout effect on the interaction of mouse gut mucosa and microbial communities in mice on the diet supplemented with amino acids, which are substrates for trace amine production, still needs further investigation. Another significant limitation is associated with the limited capacity of RNA sequencing for gene expression identification. The resulting level of gene expression depends on multiple factors, including RNA stability and modifications, the translation rate, and protein turnover, but RNA sequencing revealed only the known RNA levels in the sample. Also, we have limited options for the comparative study of TAAR1 expression in public transcriptomic data because of the small number of available datasets. Furthermore, we did not experimentally validate our investigation of TAAR1 expression in the gut wall cell populations, as this was beyond the scope of this study given that the literature [3,4,5,6,7,8] already established this point. This work focused on analyzing the function of genes co-expressed with TAAR1 through computational methods, and its results are preliminary and need a complex approach for further evaluation, which may be a subject for future studies.

Despite the limitations described above, we identified some associations between TAAR1 expression and other genes, which may exemplify this receptor participation in gastrointestinal tract functions. TAAR1 is co-expressed with Serpina1 in enteroendocrine cells, which allows us to suggest that this receptor, which seems to be commonly expressed in this cell type, is upregulated in maturating and premature enteroendocrine cells, which raises the question of the effect of amino acid derivatives on enteroendocrine cell development and further fate. The TAAR1 co-expression with calbindin in mesenteric neurons points to an uneven distribution of TAAR1 expression in this complex cell group and possible preferable expression in sensory neurons. The limited volume of data does not allow us to suggest the precise mechanism by which TAAR1 may influence the microbial community. However, we demonstrated that its loss leads to stabilizing fecal microbiota composition and a slight increase in bacterial biodiversity of fecal samples.

## 4. Materials and Methods

### 4.1. Data Collection and Inclusion Criteria for Datasets

The expression profiles of the colon tissue were received from publicly available transcriptome datasets. RNA sequencing data were searched in the National Center of Biotechnology Information (NCBI) Gene Expression Omnibus (GEO) [71]. RNAseq-generated datasets were selected that met the following criteria: (1) complete TAAR1 expression data in raw counts, FPKM, or TPM; (2) four or more biological replicates per study group; (3) mouse samples; (4) data for separated cell fractions; and (5) because of low TAARs mRNA transcription levels, for whole tissue samples, only with a minimum number of reads in SRA files > 10 million spots. After excluding irrelevant data, five datasets generated by RNAseq were included in the review (Table 1).

### 4.2. Data Normalization and Statistical Analysis

Raw RNAseq counts were downloaded and normalized to counts per million (CPM). CPM values above the threshold level of 0.1 were considered positive. The distribution of CPM-normalized expression levels in the analyzed samples was visualized using the ggplot2 v.3.5.1 R package [72].

The differentially expressed genes were identified after the trimmed mean of *M*-value normalization using a likelihood ratio test with edgeR v4.2.1 R package [72]. *p* values were adjusted for multiple testing corrections using the Benjamini–Hochberg method. Genes were considered differentially expressed if adjusted *p* values (*Pp*_adj_) < 0.05.

### 4.3. Gene Co-Expression Measurement and Pathway Enrichment Analysis

Data for different study groups were analyzed independently. TAAR1 co-expressed genes were selected using Spearman’s correlation coefficient (ρ > 0.7, *p* < 0.05). For large gene clusters, the top 50 TAAR1 co-expressed genes were selected using Spearman’s correlation coefficient value.

GO and KEGG pathway enrichment analysis (identification of GO terms and KEGG pathway components, which are significantly enriched by the genes of the selected set, respectively) was performed using clusterProfiler v.4.12.6 R package [73]. For the GO enrichment test, the biological process (BP) and molecular function (MF) ontologies were applied. Visualization of results was performed using the enrichplot v.1.24.4 R package.

### 4.4. Animals and Sample Collection

All animal studies were carried out according to the Ministry of Health of Russian Federation guidelines and the principles adopted by the FELASA and RusLASA organizations’ welfare for laboratory animal use. All experiments were approved by the Saint Petersburg State University Ethical Committee for Animal Research (no. 131-03-1 of 13.03.2022). TAAR1 knockout mice (TAAR1-KO; a generous gift of H. Lundbeck A/S) were developed by Xenogen Corporation (Cranbury, NJ, USA). In these animals, TAAR1 exon was replaced with a neo-cassette by homologous recombination as described previously [73]. All mice were *genotyped* by PCR using *ear biopsy* genomic DNA [14,73]. Wild-type (WT) and TAAR1-KO mice were derived by crossing (over 20 generations) heterozygous TAAR1 C57BL6/129SvJ animals. Experimental male mice (12 weeks old) were housed 3–5/cage, maintained under standard lab conditions (room temperature and humidity were 21 ± 5 °C and 40–70%, respectively), and provided with food and water ad libitum.

### 4.5. Gut Bacterial DNA Extraction and Sequencing

Fecal samples (100 mg) were used for DNA extraction with the Hipure Soil DNA Kit (Magen Biotech, Guangzhou, China) according to the manufacturer’s protocol. The V3-V4 region of the 16S rRNA was amplified with the sequencing primers (positions based on *E. coli* SSU rRNA numbering): 16S F 343-CCTACGGGNGGCWGCAG and 16S R 806-GGACTACHVGGGTATCTAATCC. These primers target the V3-V4 hypervariable region of bacterial 16S rRNA genes. PCR was then performed under the following conditions: denaturation (95 °C, 3 min) and then 25 cycles of denaturation (95 °C, 30 s), annealing (55 °C, 30 s), elongation (72 °C, 30 s), and final elongation (72 °C, 5 min). Sequencing was performed on the platform of Illumina HiSeq 2000 (San Diego, CA, USA).

### 4.6. Sequencing Data Processing

The raw data of 16S rRNA paired-end reads were processed using the DADA2 v.1.32.0 [74] pipeline to generate an amplicon sequence variant (ASV) table. Each ASV was annotated with the SILVA high-quality ribosomal RNA database (realize 138.2) [75].

### 4.7. Statistical Analysis of 16S rRNA Sequencing Data

α-diversity was estimated with the observed index, Chao1 estimator, abundance-based coverage estimator (ACE), Shannon index, Simpson index, and Pielou index. The above α-diversity indexes were estimated by the MicrobiotaProcess R package version 1.14.1 [76]. α-diversity index differences between genotypes were estimated with the Wilcoxon rank-sum test. Phyla abundances in studied samples were visualized with Microbiota process R package version 1.14.1 [76] and ggplot2 R package version 3.5.1. [77].

Bray–Curtis distance indexes between samples were calculated by MicrobiotaProcess R package version 1.14.1 [76] and visualized as the heatmap plot and boxplots in the same package.

Limma v.3.60.5 [77] was used to evaluate statistically significant differences in the relative abundance of operational taxonomic units (OTU) between control and TAAR1-KO samples.

The microbial co-occurrence was analyzed using cooccur v.1.3. [78] and visualized with igraph R package v.2.1.1 [79].

## 5. Conclusions

Trace amines, like PEA, TYR, and others, are present in the nervous system, where TAAR1 was described primarily [2], in extremely low concentrations. In contrast, the gut lumen content consists of higher concentrations of these compounds, which reach 1.5–4 μM/mg in feces. These molecules could activate TAAR1 receptors, stimulating the colon motility, neuropeptide Y and monoamine neurotransmitter release, and immune response. Taking into consideration the described involvement of TAAR1 in the interaction between the gut microbial community and host, we studied the impact of TAAR1 depletion on the mouse gut microbiota composition. We also analyzed TAAR1 expression in public transcriptomic data to identify its functional relations in the colonic tissue. Previously, TAAR1 expression was identified in various groups of intestinal wall cells, including the enterocytes, intestinal neuroendocrine cells, myenteric neurons, and glia. In the scope of this present study, we reanalyzed the available public transcriptomic data for the relevant cell populations and suggested the involvement of TAAR1 in enteroendocrine cell maturation and its preferential expression in the sensory neurons of the mesenteric nervous system. Likewise, we revealed TAAR1 expression in tuft cells, which simultaneously express other chemosensory GPCRs, including taste receptors. Our data of 16S rRNA sequencing in fecal samples of TAAR1-KO mice confirm TAAR1’s participation in the interaction between the microbial gut community and host. TAAR1 gene knockout leads to shifts in microbial community richness and stability. Combined with previous studies of microbiome regulation in TAAR9 knockout rats [61] and the chemosensory nature of the TAAR receptor family [1], it can be suggested that it may play an inhibitory or negative feedback role in the regulation of the microbiota composition. However, the biological processes that underpin this interaction need further investigation.

## Figures and Tables

**Table 1:** Characteristics of datasets included in the review.

Accession	Title	Cell Type	*n* *
GSE121489	Transcriptomics analysis of enteroendocrine cells after vertical sleeve gastrectomy	Enteroendocrine cells form ileum and colon	5 (ileum) and 5 (colon)
GSE124471	Bulk RNA-sequencing and Single-cell RNA-sequencing of adult mice intestinal stromal cells and lacteal LECs with PDGFRb-specific deletion of LATS1/2 [RNA-seq]	Intestinal stromal cell	6
GSE140291	Expression analysis of enteric neurons from the mice housed in different animal facilities (the Francis Crick Institute and the University of Bern)	Neurons	16
GSE143915	Transcriptomic and Proteomic Signatures of Stemness and Differentiation in the Colon Crypt	Tuft cells and enterocytes	5 tuft cells and 5 enterocyte samples
GSE151863	Impaired estrogen signaling underlies regulatory T cell loss-of-function in the chronically-inflamed intestine	Regulatory T cells	6
GSE182252	Transcriptomic analysis of the follicle associated epithelium of Peyer’s Patches, intestinal villous epithelium and ileum from young and aged mice	Enterocytes and epithelium of Peyer’s Patches	Follicle associated epithelium (*n* = 3 mice), intestinal and ileum epithelium (*n* = 4–6 mice)

* The number of controls and wild-type and non-treated samples included in the analysis.

## Data Availability

The data presented in this study are available in [repository name] at [URL/DOI], reference number [reference number]. These data were derived from the following resources available in the public domain: [list resources and URLs]. [Transcriptomics analysis of enteroendocrine cells after vertical sleeve gastrectomy] https://www.ncbi.nlm.nih.gov/geo/query/acc.cgi?acc=GSE121490] (accessed on 24 September 2024) [GSE121490] [Bulk RNA-sequencing and Single-cell RNA-sequencing of adult mice intestinal stromal cells and lacteal LECs with PDGFRb-specific deletion of LATS1/2 [RNA-seq]] [https://www.ncbi.nlm.nih.gov/geo/query/acc.cgi?acc=GSE124471] (accessed on 20 September 2024) [GSE124471] [Expression analysis of enteric neurons from the mice housed in different animal facilities (the Francis Crick institute and the University of Bern)] [https://www.ncbi.nlm.nih.gov/geo/query/acc.cgi?acc=GSE140291] (accessed on 24 September 2024) [GSE140291] [Transcriptomic and Proteomic Signatures of Stemness and Differentiation in the Colon Crypt] [https://www.ncbi.nlm.nih.gov/geo/query/acc.cgi?acc=GSE143915] (accessed on 26 September 2024) [GSE143915] [Impaired estrogen signaling underlies regulatory T cell loss-of-function in the chronically-inflamed intestine] [https://www.ncbi.nlm.nih.gov/geo/query/acc.cgi?acc=GSE151863] (accessed on 24 September 2024) [GSE151863].

## Supplementary Materials

The following supporting information can be downloaded at: https://www.mdpi.com/article/10.3390/ijms252313216/s1.
