# Supplementary material for: Functional Analysis of TAAR1 Expression in the Intestine Wall and the Effect of Its Gene Knockout on the Gut Microbiota in Mice
Source: Int J Mol Sci. 2024 Dec 9;25(23):13216. doi: 10.3390/ijms252313216 (PMC11642329; doi:10.3390/ijms252313216)
Supplement: Supplementary file 1 [file ijms-25-13216-s001.zip › ijms-3296607-supplementary.pdf]

SUPPLEMENTARY

Table S1. Genes co-expressed with TAAR1 in the enteroendocrine cells

**Pasted**	**Symbol**	**Type**	**Description**
20700	*Serpina1a*	coding	serine (or cysteine) peptidase inhibitor, clade A, member 1A
76220	*6530402F18Rik*	lncRNA	RIKEN cDNA 6530402F18 gene
71208	*4933440M02Rik*	lncRNA	RIKEN cDNA 4933440M02 gene
72669	*2810032G03Rik*	lncRNA	RIKEN cDNA 2810032G03 gene
74396	*4933407K13Rik*	lncRNA	RIKEN cDNA 4933407K13 gene
16174	*Il18rap*	coding	interleukin 18 receptor accessory protein
67198	*Spats2l*	coding	spermatogenesis associated, serine-rich 2-like
23792	*Adam23*	coding	a disintegrin and metallopeptidase domain 23
227326	*Gpr55*	coding	G protein-coupled receptor 55
76969	*Chst1*	coding	carbohydrate sulfotransferase 1
17754	*Map1a*	coding	microtubule-associated protein 1 A
18741	*Pitx2*	coding	paired-like homeodomain transcription factor 2
17389	*Mmp16*	coding	matrix metallopeptidase 16
384009	*Glipr2*	coding	GLI pathogenesis-related 2
54357	*Epb41l4b*	coding	erythrocyte membrane protein band 4.1 like 4b
231004	*Samd11*	coding	sterile alpha motif domain containing 11
242819	*Rundc3b*	coding	RUN domain containing 3B
71116	*Stx18*	coding	syntaxin 18
58805	*Mlxipl*	coding	MLX interacting protein-like
58229	*Efcc1*	coding	EF hand and coiled-coil domain containing 1
213522	*Plekhg6*	coding	pleckstrin homology domain containing, family G (with RhoGef domain) member 6
20678	*Sox5*	coding	SRY (sex determining region Y)-box 5
57278	*Bcam*	coding	basal cell adhesion molecule
233332	*Adamts17*	coding	a disintegrin-like and metallopeptidase (reprolysin type) with thrombospondin type 1 motif, 17
22141	*Tub*	coding	tubby bipartite transcription factor

67375	*Qprt*	coding	quinolinate phosphoribosyltransferase
237558	*Myrfl*	coding	myelin regulatory factor-like
20564	*Slit3*	coding	slit guidance ligand 3
19193	*Pipox*	coding	pipecolic acid oxidase
21833	*Thra*	coding	thyroid hormone receptor alpha
22415	*Wnt3*	coding	wingless-type MMTV integration site family, member 3
217721	*Flvcr2*	coding	feline leukemia virus subgroup C cellular receptor 2
20701	*Serpina1b*	coding	serine (or cysteine) preptidase inhibitor, clade A, member 1B
20702	*Serpina1c*	coding	serine (or cysteine) peptidase inhibitor, clade A, member 1C
20704	*Serpina1e*	coding	serine (or cysteine) peptidase inhibitor, clade A, member 1E
75731	*Idnk*	coding	idnK gluconokinase homolog (*E. coli*)
67392	*4833420G17Rik*	coding	RIKEN cDNA 4833420G17 gene
12289	*Cacna1d*	coding	calcium channel, voltage-dependent, L type, alpha 1D subunit
219103	*Cenpj*	coding	centromere protein J
11514	*Adcy8*	coding	adenylate cyclase 8
56722	*Litaf*	coding	LPS-induced TN factor
207227	*Stxbp5l*	coding	syntaxin binding protein 5-like
207798	*Gramd1c*	coding	GRAM domain containing 1C
12013	*Bach1*	coding	BTB and CNC homology 1, basic leucine zipper transcription factor 1
20609	*Sstr5*	coding	somatostatin receptor 5
69097	*Trim15*	coding	tripartite motif-containing 15
110855	*Pde6c*	coding	phosphodiesterase 6C, cGMP specific, cone, alpha prime
20411	*Sorbs1*	coding	sorbin and SH3 domain containing 1
245404	*Dcaf12l1*	coding	DDB1 and CUL4 associated factor 12-like 1
110954	*Rpl10*	coding	ribosomal protein L10

Table S2. Genes co-expressed with TAAR1 in the enteric neurons

Entrez ID	Gene symbol	Type	Name
77124	*9130221H12Rik*	lncRNA	RIKEN cDNA 9130221H12 gene
20341	*Selenbp1*	coding	selenium binding protein 1
66185	*Virma*	coding	vir like m6A methyltransferase associated
12307	*Calb1*	coding	calbindin 1
258331	*Olfr1330*	coding	olfactory receptor 1330
19692	*Reg1*	coding	regenerating islet-derived 1
654801	*Zfp784*	coding	zinc finger protein 784
109359	*Abraxas2*	coding	BRISC complex subunit
212531	*Sh3bgrl2*	coding	SH3 domain binding glutamic acid-rich protein like 2
216393	*D930020B18Rik*	coding	RIKEN cDNA D930020B18 gene
258068	*Olfr804*	coding	olfactory receptor 804
21452	*Tcn2*	coding	transcobalamin 2
12467	*Cct6b*	coding	chaperonin containing Tcp1, subunit 6b (zeta)
66895	*Pxdc1*	coding	PX domain containing 1
13025	*Ctla2b*	coding	cytotoxic T lymphocyte-associated protein 2 beta
28113	*Tinf2*	coding	Terf1 (TRF1)-interacting nuclear factor 2
29820	*Tnfrsf19*	coding	tumor necrosis factor receptor superfamily, member 19
68705	*Gtf2f2*	coding	general transcription factor IIF, polypeptide 2
58245	*Gpr180*	coding	G protein-coupled receptor 180
22337	*Vdr*	coding	vitamin D (1,25-dihydroxyvitamin D3) receptor
77652	*Zfp955a*	coding	zinc finger protein 955A
260409	*Cdc42ep3*	coding	CDC42 effector protein (Rho GTPase binding) 3
240327	*Gm4951*	coding	predicted gene 4951
100416240	NA	NA	NA
100417831	NA	NA	NA
545472	NA	NA	NA

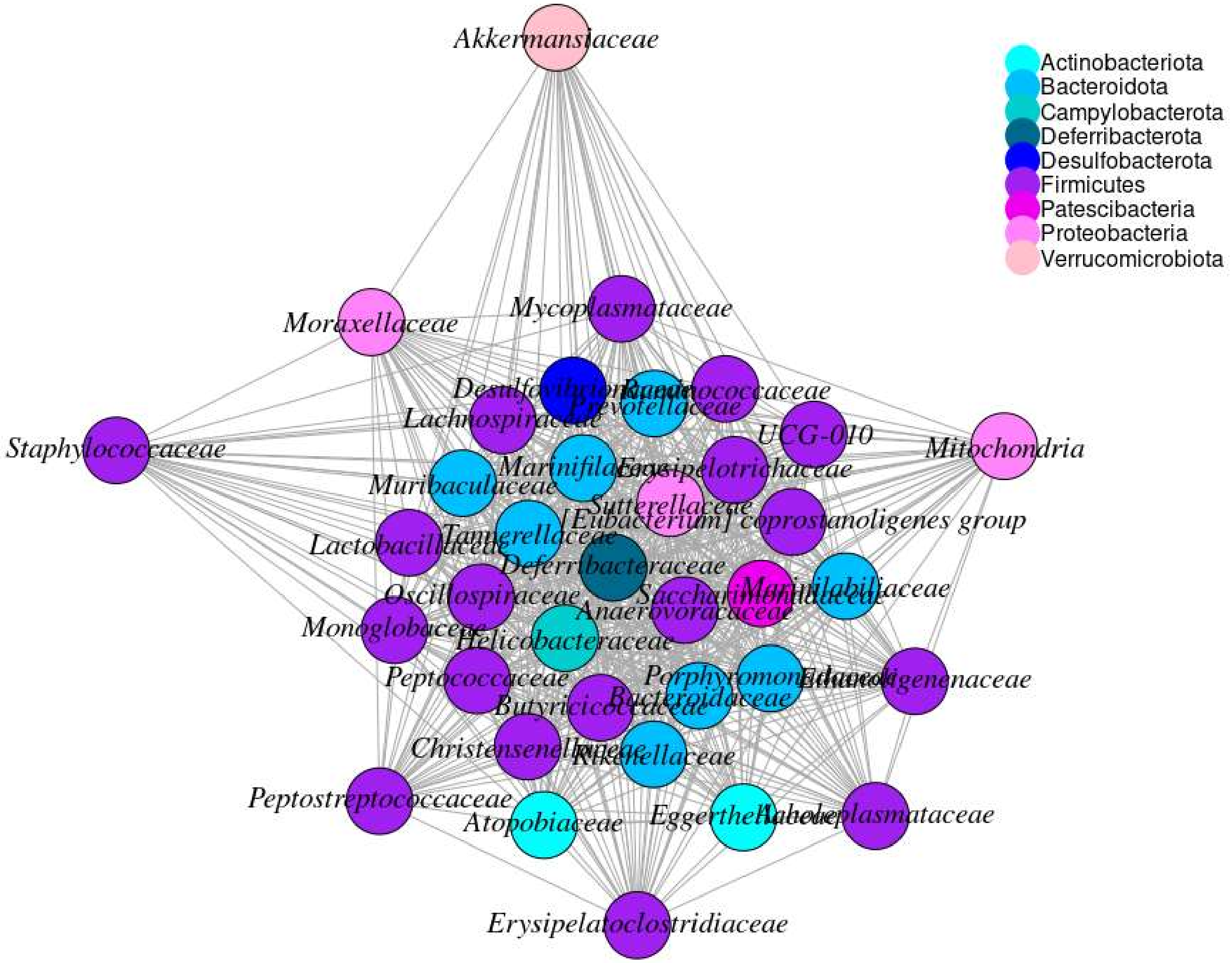

Figure S1. Bacterial families' co-occurrence network in WT mice fecal samples. The nodes represent families, the edge's length demonstrates frequency of co-occurrence (longer distance for rarely co-occurred families and closer distance for frequently co-occurred).

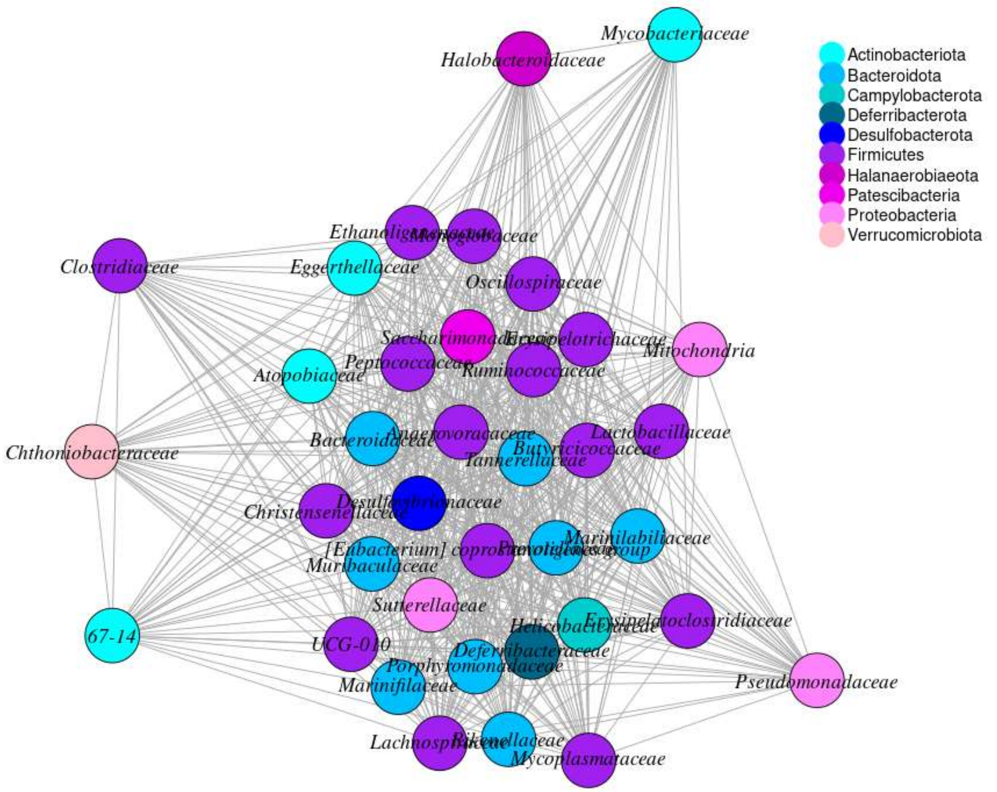

Figure S2. Bacterial families' co-occurrence network in TAAR1-KO mice fecal samples. The nodes represent families, the edge's length demonstrates frequency of co-occurrence (longer distance for rarely co-occurred families and closer distance for frequently co-occurred).
